# Management of Emerging Health Conditions to Improve Resilience and Mental Health

**DOI:** 10.3390/healthcare10101908

**Published:** 2022-09-29

**Authors:** Yi-Chih Lee, Wei-Li Wu

**Affiliations:** Department of International Business, Chien Hsin University, Taoyuan 320312, Taiwan

Good health is when a person is in a complete, optimal physical, mental, and social condition. Countries take different measures to promote health and control diseases. It is of great significance and value to promote medical, psychological, and public health to ensure good health among populations [1,2]. The public’s right to know and active cooperation are crucial to improving public health [3].

Mental health has intrinsic and instrumental value and is a state of mental health that is determined by the interaction of individual, social, and structural stresses. Mental health enables people to cope with the stresses of life, perform their jobs well, develop their abilities, and contribute to organizations and society. It is also an indispensable element of health and well-being. Exposure to adverse organizational, social, economic, geopolitical, and external environments increases people’s risk of developing mental health problems. Therefore, promoting people’s mental health through various channels and providing supportive services to people with mental health problems can prevent their human rights violation [4,5].

In recent years, humans have been fighting against emerging infectious diseases, such as COVID-19, which broke out in 2019 [6], and monkeypox, in 2022 [7]. The spread of viruses significantly impacts public health and medical care worldwide. Regarding the physical and mental issues faced by frontline medical workers, positive mental training may be used to improve their ability to regulate their emotions [8]. The pandemic has affected the sustainability of public environments and introduced health problems [9]. Distance learning and working have led to changes in health behaviors [10,11]. Furthermore, people need to face organizational adaptation [12] and concerns for self-health [13] when fighting against diseases. These changes indicate that emerging diseases systematically impact human society and require attention from all facets of society.

Public health issues have been progressing globally. Scholars interested in topics such as psychology, public health, and alleviating the impact of various emerging diseases on global health are welcome to submit manuscripts to the Special Issue “Management in Different Health Conditions”.

## Data Availability

Not applicable.
